# Synergy Between the Auranofin Analogue PEt_3_AuCl and Membrane Disruptors, Efflux-Pump Blockers, and Glutathione-Depletors Uncovers Tolerance Pathways in *Pseudomonas aeruginosa*

**DOI:** 10.3390/ijms27104610

**Published:** 2026-05-21

**Authors:** Beatrice Amato, Diletta Mazzantini, José Aleixo de Azevedo-França, Giuseppantonio Maisetta, Emilia Ghelardi, Semih Esin, Luigi Messori, Giovanna Batoni

**Affiliations:** 1 Department of Translational Research and New Technologies in Medicine and Surgery, University of Pisa, 56126 Pisa, Italy; beatrice.amato@med.unipi.it (B.A.); diletta.mazzantini@unipi.it (D.M.); giuseppantonio.maisetta@unipi.it (G.M.); emilia.ghelardi@unipi.it (E.G.); semih.esin@unipi.it (S.E.); 2 Department of Chemistry, University of Florence, 50019 Florence, Italy; josealeixo.deazevedofranca@unifi.it (J.A.d.A.-F.); luigi.messori@unifi.it (L.M.)

**Keywords:** auranofin, auranofin analogue, PEt_3_AuCl, gold conjugates, drug repurposing, *Pseudomonas aeruginosa*, tolerance mechanisms

## Abstract

Auranofin (AF), an FDA-approved drug for rheumatoid arthritis, exhibits strong antibacterial activity against Gram-positive bacteria, while Gram-negative species remain largely tolerant. This study assessed the antimicrobial activity of AF and three analogues against clinically relevant Gram-negative pathogens and explored tolerance mechanisms in *Pseudomonas aeruginosa*. Broth microdilution assays were performed on reference strains and clinical isolates of *Escherichia coli*, *Klebsiella pneumoniae*, and *P. aeruginosa*. Synergy studies with the most active analogue, PEt_3_AuCl (AF-Cl), were conducted against *P. aeruginosa* using polymyxin B (PMB), two efflux-pump inhibitors, and two glutathione (GSH) depletors. Gold compounds showed MICs between 4 and >64 µg/mL, with AF-Cl displaying the highest activity. AF-Cl activity was markedly enhanced by PMB and efflux-pump inhibitors, indicating that outer membrane permeability and efflux contribute to tolerance. Additionally, GSH depletion significantly potentiated AF-Cl, implicating redox homeostasis in resistance. Overall, AF-Cl shows potential against Gram-negative bacteria when combined with agents targeting membrane integrity, efflux systems, or redox balance, supporting combinatorial strategies to overcome resistance in *P. aeruginosa* and related pathogens.

## 1. Introduction

Multidrug-resistant Gram-negative bacterial (MDR-GNB) infections pose a serious threat to human health due to the limited therapeutic options currently available [1]. Drug repurposing has emerged as a promising strategy to address these treatment challenges [2]. Notably, drugs originally approved for non-antimicrobial indications may exhibit antimicrobial properties. These compounds can act as standalone antimicrobial agents, enhance the efficacy of existing antibiotics, or serve as molecular scaffolds for the development of derivatives with improved antimicrobial activity [3].

Recent studies have highlighted the antimicrobial potential of auranofin (AF), a gold-based drug approved for the treatment of rheumatoid arthritis, which shows particular efficacy against Gram-positive bacteria [4]. AF acts by inhibiting thioredoxin reductase (TrxR), a key enzyme involved in maintaining cellular redox homeostasis. This disruption leads to oxidative stress, ultimately causing cell death in both pathogens and cancer cells [4]. Inhibition of TrxR by AF is primarily attributed to direct association of gold with functional cysteines in the active site of the enzyme [4]. Additionally, AF has been reported to interfere with several biosynthetic pathways, including DNA, RNA, and protein synthesis, as well as cell wall formation—at least in Gram-positive bacteria [5]. This multi-targeted mechanism of action may reduce the likelihood of resistance development compared to conventional antibiotics. Furthermore, AF’s ability to inhibit biofilm formation and inflammatory pathways [6,7,8,9] makes it a promising candidate for treating chronic biofilm-associated infections, which are often refractory to therapy and accompanied by severe inflammation and tissue damage.

Although AF exhibits strong activity against Gram-positive bacteria at nano - to micromolar concentrations, its efficacy against Gram-negative bacteria is significantly lower, limiting its potential as a broad-spectrum antimicrobial agent [5,10]. Several hypotheses have been proposed to account for the reduced susceptibility of Gram-negative bacteria to AF. These include (i) the barrier effect played by the Gram-negative outer membrane; (ii) the presence of efflux pumps, primarily expressed in Gram-negative bacteria; (iii) the high intracellular reserves of glutathione (GSH), one of the major redox buffer and detoxifying agents of Gram-negative bacteria. For instance, an increase in susceptibility of Gram-negative species to AF has been reported when the compound was combined with colistin, a membrane-permeabilizing antibiotic [10]. Other studies, based on the use of mutant strains of the model bacterium *Escherichia coli* support a role for efflux pumps in attenuating AF antimicrobial effectiveness towards Gram-negative bacteria. For instance, an *E. coli* strain with deletion of the membrane protein TolC, which is responsible for the extrusion of several antibiotics and metal ions, was found to be slightly more susceptible to AF than the corresponding wild-type strain [11]. Similar results were obtained when the efflux pump AcrAB was deleted in *E. coli* [5]. Finally, a recent study demonstrated a marked increase in susceptibility to AF of an *E. coli* mutant strain lacking ϒ-glutamate-cysteine-ligase (GsHA). This enzyme catalyzes the first step in the synthesis of glutathione (GSH), an antioxidant present in many Gram-negative bacteria, but absent in many Gram-positive bacteria. This suggests that in *E. coli*, intracellular reserves of GSH may compensate for AF-induced impairment of the Trx system and play a significant role in AF resistance [11].

Despite these studies, the precise underlying mechanisms of AF resistance/tolerance in Gram-negative bacteria remain incompletely understood, and further research is needed to assess the exact role of the different contributors in the development of AF resistance/tolerance in clinically relevant species.

The aim of this work was to identify AF analogues with enhanced activity against Gram-negative species. In addition, through synergistic combinations we sought to uncover potential defense mechanisms that could mediate the tolerance pathways of *Pseudomonas aeruginosa* towards one of these analogues (PEt_3_AuCl–AF-Cl). This bacterium is a high-priority species according to the WHO, intrinsically resistant to many antibiotics, and the leading cause of hospital-acquired infections [12]. The results obtained demonstrated that AF-Cl was the most active AF analogue with a 2–8 fold reduction in the MIC values as compared to the parental AF. Furthermore, AF-Cl activity against *P. aeruginosa* was highly enhanced in combination with the membrane-permeabilizing agent polymyxin B (PMB), and with two efflux-pump inhibitors, carbonyl cyanide-m-chlorophenylhydrazone (CCCP) and phenylalanine-arginine β-naphthylamide (PaβN). These results suggest that the relatively low activity of AF-Cl against *P. aeruginosa* may be due to a combination of the barrier effect of the outer membrane and extrusion via efflux pumps. Furthermore, AF-Cl exhibited significantly enhanced antimicrobial activity in combination with GSH depletors (L-buthionine sulfoximine–BSO- and diethyl maleate –DEM- or both), which suggests also a role of GSH in *P. aeruginosa* tolerance pathways to AF-Cl. These findings imply that combinatorial strategies based on the improved AF analogue AF-Cl may offer new possibilities for therapeutic interventions against resistant Gram-negative infections.

## 2. Results

### 2.1. Determination of the MIC of AF and Its Analogues Against Gram-Negative Bacteria

The minimum inhibitory concentrations (MICs) of AF and its three analogues (AF-Cl, PEt_3_AuI [AF-I], and PPh3AuCl [TPP-AuCl]) were determined using the standard broth microdilution method against reference strains and clinical isolates of Gram-negative bacteria. As reported in Table 1, the MIC values of AF ranged from 32 to >64 μg/mL. These values are substantially higher than those previously reported for Gram-positive species by our group [13] and other investigators [5,10]. The analogue AF-Cl demonstrated a two- to eight-fold reduction in MIC values across the same strain panel. For most strains tested (five out of nine), AF-I displayed MIC values exceeding those of AF-Cl for the corresponding isolates. TPP-AuCl was the least active analogue, with MIC values > 64 μg/mL for all strains except *E. coli* ATCC 25922. Based on these findings and previous research indicating that AF-Cl is the least toxic to human cells [13], AF-Cl was identified as the most promising compound for further investigation.

### 2.2. Synergistic Effect of AF-Cl with the Membrane-Permeabilizing Agent PMB

**Figure 1:**
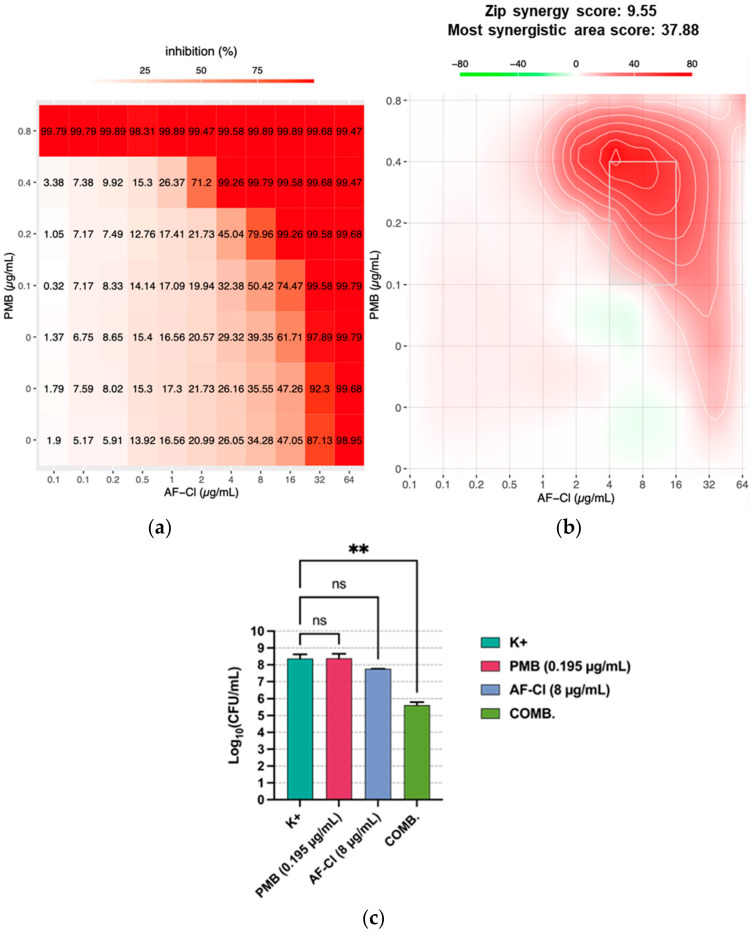
Synergistic effect of AF-Cl with the membrane-permeabilizing agent PMB against *P. aeruginosa* ATCC 15442. (**a**) Computer-assisted analysis (SynergyFinder) of the dose–response inhibition matrix and (**b**) of the most synergistic area obtained in a representative experiment. (**c**) Synergistic effect evaluated as CFU count: the panel depicts the mean values ± SEM of two separate experiments each conducted in duplicate; K+: bacteria incubated in medium only; PMB: polymyxin B; COMB.: combination; ns: non-statistically significant; and ** *p* < 0.01, one-way ANOVA.

In order to explore whether the outer membrane could play a role in the relatively low susceptibility of Gram-negative species to AF-Cl, synergistic assays were performed between AF-Cl and the membrane-permeabilizing agent PMB against *P. aeruginosa*, a medically relevant Gram-negative bacterium, which has emerged as one of the least susceptible species to AF-Cl. A total of 77 combinations of the two compounds were tested by a standard checkerboard assay and the results analyzed in terms of dose–response inhibition, synergy score and most synergistic area using the computational tool SynergyFinder 3.0 [14,15]. As shown in the dose–response matrix for the *P. aeruginosa* ATCC 15442 strain (Figure 1a), the concentrations of both AF-Cl and PMB required to inhibit over 98% of bacterial growth decreased when the two compounds were combined, compared to when they were used individually. Figure 1b depicts the combinations that showed maximal synergy at lower doses (the dotted box) and the summary synergy score—averaged over all of the dose combination measurements. The latter was 9.556, which is very close to the cut-off for synergy, increasing to 37.88 in the most synergistic area. The likely synergistic effect was also confirmed for the ATCC *P. aeruginosa* strain in terms of CFUs surviving the treatment. As shown in Figure 1c, combining AF-Cl at the concentration of 8 μg/mL (1/8 MIC) with PMB at the concentration of 0.195 μg/mL (1/4 MIC) reduced the CFU count by ≥2 log_10_ units (i.e., 100-fold) compared to the most active single agent used alone, indicating a synergistic effect of this combination.

For both the ATCC strain and two *P. aeruginosa* clinical isolates, the results were also confirmed in terms of FICi values that were ≤0.5, consistent with positive interactions between the two drugs (Table 2). Information on the directionality and nature of the AF-Cl/PMB interaction was investigated considering the individual FICs as separate metrics [16] (Table 2). For all of the combinations, FIC_PMB→AF-Cl_ and FIC_AF-CL→PBM_ were ≤0.25, suggesting that each compound promotes the action of the other.

### 2.3. Synergistic Effect of AF-Cl and Efflux-Pump Inhibitors

**Figure 2:**
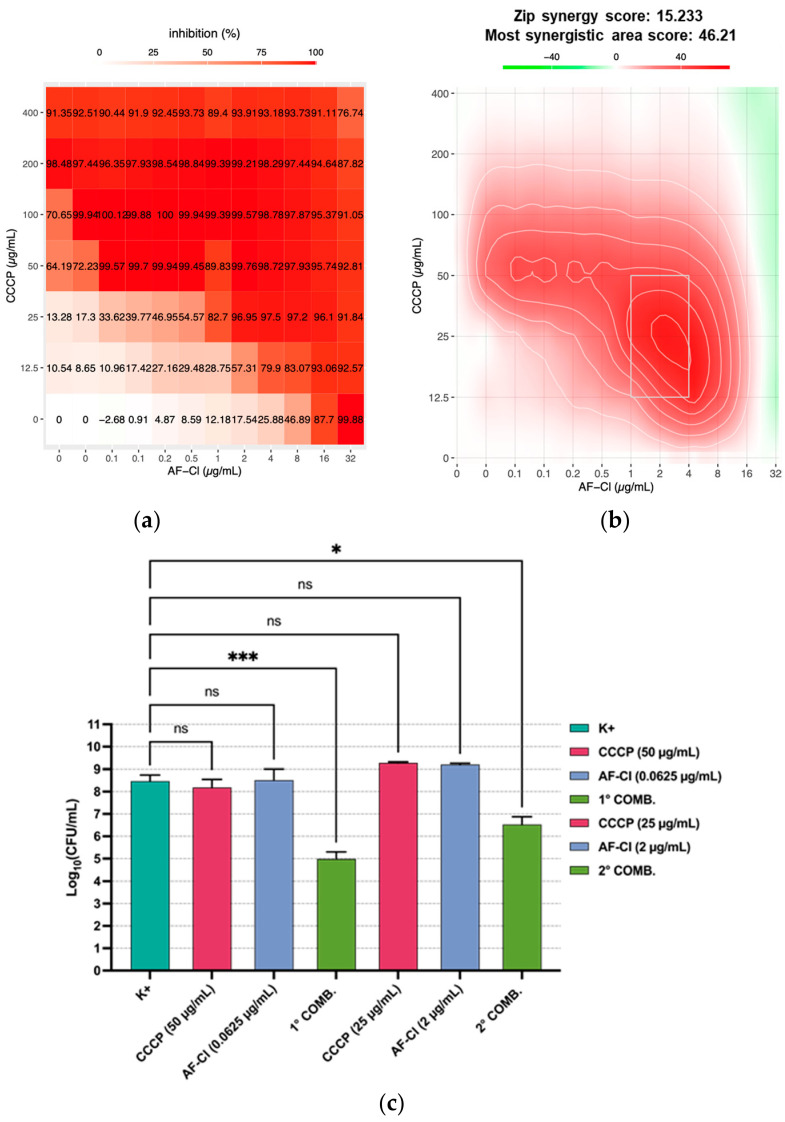
Synergistic effect of AF-Cl with the EPI CCCP against *P. aeruginosa* ATCC 15442. (**a**) Computer-assisted analysis (SynergyFinder) of the dose–response inhibition matrix and (**b**) of the most synergistic area obtained in a representative experiment. (**c**) Synergistic effect evaluated as CFU count: the panel depicts the mean values ± SEM of two separate experiments each conducted in duplicate; K+: bacteria incubated in medium only; CCCP: carbonyl cyanide m-chlorophenylhydrazone; COMB.: combination; ns: non-statistically significant; and * *p* < 0.05 and *** *p* < 0.001, one-way ANOVA.

**Figure 3:**
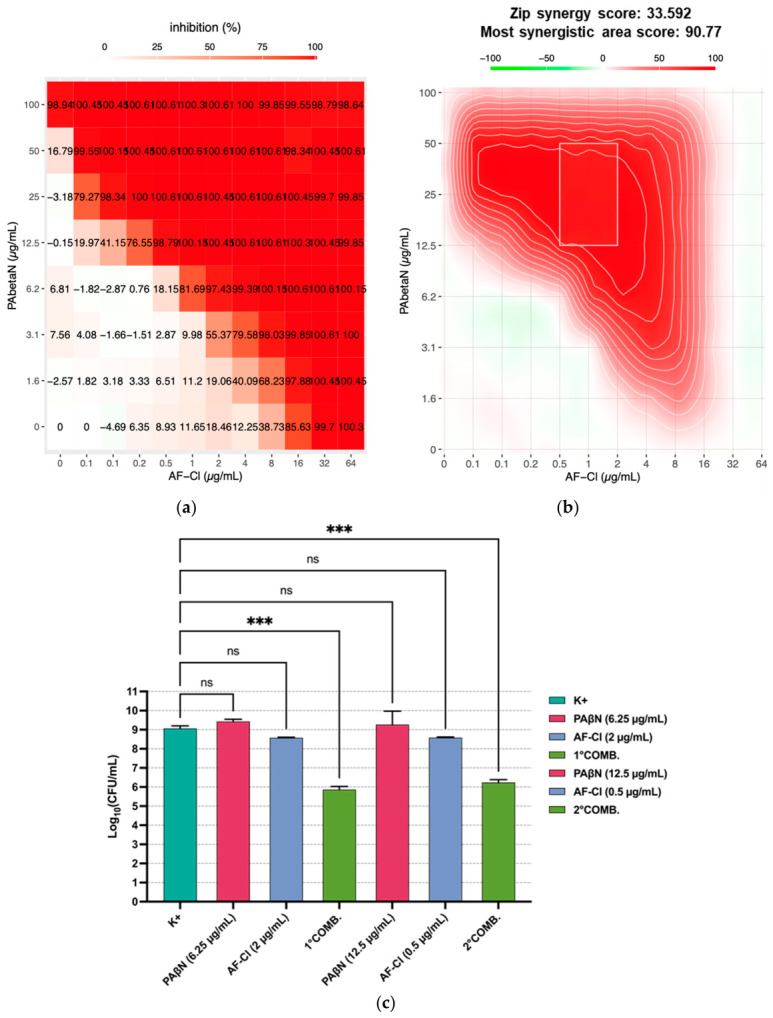
Synergistic effect of AF-Cl with the EPI PaβN against *P. aeruginosa* ATCC 15442. (**a**) Computer-assisted analysis (SynergyFinder) of the dose–response inhibition matrix and (**b**) of the most synergistic area obtained in a representative experiment. (**c**) Synergistic effect evaluated as CFU count; the panel depicts the mean values ± SEM of two separate experiments each conducted in duplicate; K+: bacteria incubated in medium only; PAβN: phenylalanine-arginine beta-naphthylamide; COMB.: combination; ns: non-statistically significant; and *** *p* < 0.001, one-way ANOVA.

As efflux pumps play a major role in reducing antibiotic effectiveness in Gram-negative bacteria, the possible effect of efflux-pump inhibitors (EPIs) in increasing the susceptibility of *P. aeruginosa* to AF-Cl was investigated, following the same procedure described in Section 2.2. For both the EPIs tested (i.e., CCCP and PaβN), an evident synergistic effect was observed when they were combined with sub-MIC concentrations of AF-Cl, with synergy scores far exceeding the cut-off of 10, especially for the EPI PAβN (Figure 2a,b and Figure 3a,b). The synergistic effect of both EPIs with AF-Cl was confirmed in terms of CFU counts for two different combinations tested (Figure 2c and Figure 3c).

FICis were consistently lower or equal to 0.5 (Table 3 and Table 4), further supporting the synergistic effect. Interestingly, analyzing the individual FICis as separate metrics showed that although all the values were ≤0.25, indicating a reciprocal positive interaction, for seven out of ten combinations tested, the FIC_EPI→AF-Cl_ was lower than the corresponding FIC _AF-Cl→EPI_. This suggests a greater contribution of EPIs in enhancing AF-Cl antipseudomonal activity than vice versa.

### 2.4. Antibacterial Effects of AF-Cl Combined with GSH Depletors

**Figure 4:**
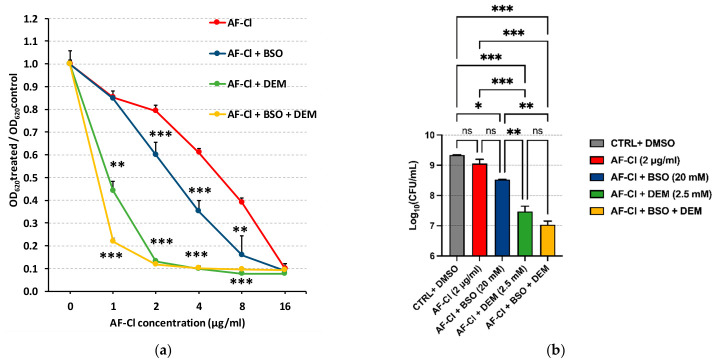
Antibacterial effects of AF-Cl on *P. aeruginosa* ATCC 15442 in conditions of GSH depletion. (**a**) Inhibition of growth evaluated as normalized OD_620_; (**b**) killing effect evaluated as CFU. This figure depicts the mean values ± SEM of two separate experiments each conducted in duplicate. CTRL: bacteria incubated in medium with solvent (DMSO) added; BSO: L-Buthionine sulfoximine 20 mM; DEM: Diethyl maleate 2.5 mM; ns: non-statistically significant; and * *p* < 0.05, ** *p* < 0.01, and *** *p* < 0.001, one-way ANOVA.

Gram-negative bacteria, including *P. aeruginosa*, employ the glutathione (GSH) system to mitigate oxidative stress generated by both host immune defenses and certain antimicrobial agents [17]. This GSH-dependent pathway could partially compensate for the oxidative imbalance resulting from TrxR inhibition, which has recently been identified as a potential molecular target of AF-Cl [18]. To evaluate this hypothesis, antibacterial assays were conducted against *P. aeruginosa* by combining AF-Cl with two GSH-depleting agents, BSO and DEM, used at the sub-MIC concentrations of 20 mM and 2.5 mM, respectively. As shown in Figure 4a, the combination of AF-Cl (2, 4, and 8 µg/mL) with BSO resulted in a statistically significant reduction in normalized OD_620_ compared with AF-Cl monotherapy. The potentiating effect on AF-Cl antibacterial activity was even more pronounced when combined with DEM or with both GSH-depleting agents. Under these conditions, an AF-Cl concentration as low as 1 µg/mL was sufficient to produce a statistically significant decrease in normalized OD_620_ relative to AF-Cl alone (*p* < 0.01 for AF-Cl versus AF-Cl + DEM; *p* < 0.001 for AF-Cl versus AF-Cl + DEM + BSO). These results were confirmed in terms of CFU count. When the triple combination was tested at an AF-Cl concentration of 2 µg/mL, a reduction of 2 log_10_ units was observed compared to AF-Cl alone (Figure 4b).

## 3. Discussion

There is widespread agreement that the rate of development of new antibiotics is fully inadequate to address the mounting threat of antibiotic resistance, especially against priority pathogens like *P. aeruginosa* [19]. Therefore, the identification of alternative anti-bacterial strategies is an expanding area of research, aiming to alleviate the pressure driving antibiotic overuse and to provide options when antibiotics become completely ineffective. Gold compounds like AF have attracted considerable interest as potential weapons against resistant bacteria due to several promising features—i.e., relevant antibacterial activity against MDR strains in vitro and in vivo; no resistance induction; activity against biofilms; and synergistic microbicidal effect with conventional antibiotics [20,21]. Nevertheless, AF possess significant cytotoxicity against eukaryotic cells in vitro and displays low activity against Gram-negative bacteria encouraging the design of analogues with improved therapeutic potential. In this study, we tested three AF analogues against a panel of Gram-negative strains and aimed to explore the mechanisms of tolerance of *P. aeruginosa* to one of these analogues through synergistic combinations. The three analogues structurally differ from AF in the groups attached to the gold atom: in AF-Cl and AF-I, the thiosugar ligand of AF is replaced by a chloride or iodide ligand, respectively. In TPP-AuCl, the gold (I) center is linearly coordinated to triethylphosphine and chlorid, respectively [13]. Both AF-Cl and AF-I displayed stronger activity compared to AF with a two- to eight-fold reduction in MIC values, although AF-l displayed MIC values exceeding those of AF-Cl for most of the isolates tested. In contrast, TPP-AuCl was markedly less active than both AF and the two analogues AF-Cl and AF-I. These results are consistent with our recent report, in which the same gold complexes were tested against a panel of Gram-positive strains, revealing the same pattern of antimicrobial activity based on MIC values: AF-Cl > AF-I > AF > TPP-AuCl [13]. Taken together, these findings support our previous hypothesis that the first two analogues interact more favorably with bacterial surfaces—of both Gram-positive and Gram-negative bacteria—leading to more efficient cellular uptake than the two latter compounds. Despite an overall reduction in the MIC values observed for AF-Cl compared to AF, they remained substantially higher if compared with those reported for Gram-positive strains, prompting further investigations to explore this aspect.

Several hypotheses have been made to explain the low susceptibility of Gram-negative bacteria to AF [4,11]. These include the barrier effect played by the outer membrane as well as the activity of efflux pumps, particularly effective in Gram-negative bacteria. Both of these mechanisms may prevent AF accumulation in the cytoplasm and its consequent interaction with cellular target(s). Furthermore, it has been proposed that the GSH system present in Gram-negative bacteria, but not in the Gram-positive ones, can compensate for the loss of the reducing ability of Trx, one of the best-characterized targets of AF. Building on these hypotheses, we aimed to investigate synergistic combinations of AF-Cl with selected compounds capable of elucidating the relative contribution of the various tolerance mechanisms employed by *P. aeruginosa*, which represents one of the bacterial species exhibiting the lowest susceptibility to AF-Cl.

When combined with PMB, a membrane-permeabilizing antibiotic, AF-Cl exhibited enhanced antipseudomonal activity, as assessed by both growth inhibition and bactericidal effects. Comparable results were previously obtained when AF was co-administered with either PMB or colistin [5,10], supporting the notion that the outer membrane plays a significant role in limiting the entry of both gold complexes into the bacterial cell. Interestingly, separate evaluation of the FICis indicated that the interaction between AF-Cl and PMB was likely reciprocal, with each compound enhancing the activity of the other. Both colistin and PMB exert their effects on the bacterial membrane, interacting with the lipid components of lipopolysaccharide (LPS) that become unstable, increasing bacterial membrane permeability [22]. Therefore, PMB could enhance AF-Cl activity, favoring its entrance into the cytosol. While the primary model for polymyxin’s antimicrobial activity involves the destruction of the bacterial membranes, additional mechanisms of action have been proposed, including perturbation of bacterial metabolism, leading to increased production of reactive oxygen species (ROS) [22,23,24]. Specifically, Lima et al. highlighted that ROS play a significant role in *P. aeruginosa* bacterial cell lethality, pointing to oxidative burst mechanisms related to PMB treatment [25]. Thus, inhibition of Trx by AF-Cl could render bacteria more susceptible to PMB-induced oxidative stress. Although in the 1980s polymyxins were gradually replaced by newer antibiotics due to their high nephrotoxicity and neurotoxicity, in the era of superbugs they have been re-evaluated as a last-resort treatment against MDR-GNB [22]. Specifically, nebulized PMB therapy is emerging in intensive care units for treating hospital-acquired and ventilator-associated pneumonia of which *P. aeruginosa* is one of the most common causative agents [26,27]. These findings suggest a potential application in clinical practice of combining AF-Cl with PMB in the treatment of *P. aeruginosa* lung infections, potentially allowing for lower PMB dosages and reduced systemic toxicity. Notably, both PMB and AF-Cl possess anti-inflammatory properties, a feature of particular relevance in *P. aeruginosa* pulmonary infections, which are typically characterized by intense inflammatory responses and substantial tissue damage.

Efflux pumps are widely utilized by bacteria to pump out antibiotics from within their cells, reducing the intracellular concentration of the drug and leading to resistance. Efflux pumps play a major role in the antibiotic resistance of *P. aeruginosa*, which possesses four multicomponent MDR Resistance-Nodulation-division (RND) efflux pumps, namely, MexAB-OprM, MexCD-OprJ, MexEF-OprN, and MexXYOprM [28]. Efflux-pump inhibitors (EPIs) can block these pumps, preventing antibiotic expulsion and increasing intracellular drug levels. Examples of EPIs include CCCP and PAβN [29]. The first disrupt the proton motive force across biological membranes. By doing so, it dissipates the proton gradient that is essential for various cellular processes, including the function of efflux pumps. PaβN, also known as MC-207,110, is a broad-spectrum EPI that acts on multiple RND-family efflux pumps in *P. aeruginosa* [30]. In this study, combining sub-inhibitory concentrations of AF-Cl with each of these EPIs resulted in a strong synergistic effect supporting a major role for efflux pumps in *P. aeruginosa* resistance to AF-Cl.

So far, no drug is clinically approved to inhibit efflux pumps in *P. aeruginosa* due to undesirable toxicities and inconsequential in vivo efficacy. However, this field of investigation is quite active and several clinically approved drugs have shown experimental efflux-pump-inhibiting activity against *P. aeruginosa*. For instance, a 2025 study demonstrated that FDA-approved proton pump inhibitors like omeprazole, esomeprazole, or pantoprazole can reduce expression of mexA (efflux gene) in *P. aeruginosa* and inhibit efflux activity [31]. In the same study, vitamin D and vitamin K also demonstrated strong anti-efflux activity in *P. aeruginosa* PAO1 [31]. Another study showed that repurposed drugs such as promethazine and fluoxetine (both clinically approved as antihistamines and antipsychotics, respectively) have experimental EPI activity in multidrug-resistant *P. aeruginosa* [32]. Finally, a recent report found that zinc sulfate (used in the management of diarrheal diseases as an oral supplement) inhibits efflux-pump activity in all tested *P. aeruginosa* clinical isolates and synergizes with multiple antibiotics [33]. Additionally, zinc sulfate significantly upregulated the expression of the mexR gene, encoding a negative regulator of the MexAB-OprM efflux pump. While no EPIs have yet entered clinical use, several next-generation candidates—especially those targeting Gram-negative RND pumps—are showing strong potential in preclinical studies. For example, TXA11114, which belongs to the indole-carboxamide class and was designed specifically to inhibit *P. aeruginosa* RND pumps, was shown to exhibit a safe toxicology profile and a strong in vivo efficacy when combined with levofloxacin [34].

Taken together, these findings suggest that AF-Cl/EPI combinations could be a viable therapeutic option for treating *P. aeruginosa* infections in clinical scenarios where conventional antibiotics fail to achieve eradication, and could therefore have translational potential in the near future.

It has been proposed that the glutathione system, present in Gram-negative bacteria, compensates for the loss of TrxR function, therefore reducing the efficacy of AF [5,10]. Consistent with this, Quadros Barsé and coworkers have recently demonstrated that in *E. coli*, the deletion of the gene encoding for ϒ-glutamate-cysteine-ligase (gshA), the enzyme catalyzing the first step of GSH biosynthesis, markedly enhances AF’s activity against the mutant strain (ΔgshA) compared to the wild type [11]. This suggests that the intracellular GSH pool confers *E. coli* substantial protection against oxidative stress induced by AF [11].

Glutathione is a key antioxidant in *P. aeruginosa*, helping the bacterium cope with redox stress in both environmental and host-associated conditions [17]. In order to uncover whether GSH could play a role in *P. aeruginosa* tolerance to AF-Cl, in the present study, we tested AF-Cl in combination with two GSH depletors, BSO and DEM, in dual or triple combinations. BSO acts by inhibiting GshA and therefore GSH biosynthesis, whereas DEM depletes intracellular thiols, including GSH, through direct conjugation reactions. Sub-inhibitory concentrations of both compounds, and especially of DEM, greatly enhanced the anti-pseudomonal activity of AF-Cl. As expected, the effect was even more evident in the triple combination that was able to cause a reduction in the CFU number of almost 3 log_10_ units, at an AF-Cl concentration far lower than the MIC. These findings strongly support the view that the GSH system does play a pivotal role in *P. aeruginosa* tolerance to AF-Cl and point to GSH biosynthesis as a viable drug target in combinatorial antimicrobial strategies [35].

## 4. Materials and Methods

### 4.1. Bacterial Strains and Culture Conditions

A panel of nine bacterial strains, including three reference strains (*E. coli* ATCC 25922, *K. pneumoniae* ATCC 700603, and *P. aeruginosa* ATCC 15442) and six clinical isolates, was used to assess the antimicrobial activity of AF and three other gold-containing analogs (Table 5). Clinical strains, now part of a strain collection at the Department of Translational Research and New Technologies in Medicine and Surgery, University of Pisa, were isolated at the microbiology laboratory of Pisa University’s Hospital (Italy) using standard procedures. Briefly, species identification was performed by MALDI-TOF MS (Microflex LT, Bruker Daltonics, Bremen, Germany) using the MALDI Biotyper 3.1 software suite according to the manufacturer’s instructions. Antibiotic-resistance profiles were determined by broth microdilution using ITGN10 panels (Bruker Daltonics). For preparation of stock cultures, strains were grown in Mueller–Hinton broth (MHB, Sigma-Aldrich, Milan, Italy) to the late logarithmic phase, aliquoted, and stored at −80 °C. Cultures on solid media were performed on MHB supplemented with 1.5% (*w*/*v*) bacteriological agar.

### 4.2. Gold Compounds

The structure of the gold conjugates (AF, PEt_3_AuCl [AF-Cl], PEt_3_AuI [AF-I], and PPh3AuCl [TPP-AuCl]) used in this study has been previously reported [13]. AF and AF-Cl were purchased from Sigma-Aldrich (St. Louis, MO, USA). AF-I and TPP-AuCl were synthesized as previously reported [10,36]. The compounds were dissolved in 100% dimethyl sulfoxide (DMSO, ITW Reagents, Milan, Italy) to a final concentration of 10 mg/mL, divided in aliquots, and stored at −20 °C until use. Control experiments were performed to assess the effect of DMSO on bacterial growth, demonstrating no inhibitory activity for all tested *P. aeruginosa* strains at concentrations up to 3%; in the assays, gold compounds were diluted in the culture medium and tested at concentrations of 64 µg/mL or lower. Consequently, the final DMSO concentrations in the assays were 0.64% or lower, which is well below the levels shown to inhibit bacterial growth.

### 4.3. Determination of Minimal Inhibitory Concentrations (MICs)

The susceptibility of Gram-negative strains to AF and its analogues was assessed by determining minimum inhibitory concentrations (MICs) via standard broth microdilution assays, following EUCAST guidelines [37]. Bacterial cultures were prepared from freshly isolated colonies and suspended in sterile saline to achieve a turbidity equivalent to 0.5 McFarland (≈1.5 × 10^8^ CFU/mL). The suspension was subsequently diluted 1:150 in Mueller–Hinton broth (MHB, Sigma-Aldrich), yielding a final inoculum of approximately 5 × 10^5^ CFU/well. Test compounds were dispensed into 96-well microtiter plates by two-fold serial dilution in a final volume of 100 µL/well, followed by the addition of 100 µL of bacterial inoculum (total volume 200 µL/well). Plates were incubated at 37 ± 1 °C for 16–20 h under static conditions. Bacterial growth was assessed through visual examination of wells and confirmed by optical density readings (OD_620_) nm using a microplate ELISA reader (Multiskan FC with incubator, 51119100 Thermo Fisher Scientific, Rodano, Milan, Italy). The MIC was defined as the lowest concentration of the compound that completely inhibited visible growth and that prevented any increase in OD_620_ relative to the growth control. Negative controls (wells containing uninoculated medium) and positive controls (wells containing bacterial inoculum without compounds, but with DMSO at the corresponding final concentrations) were included in each assay. All experiments were performed in triplicate.

### 4.4. Checkerboard Assays for Synergistic Studies

The interaction between AF-Cl and other potential AF-Cl-activity enhancers was assessed using a checkerboard assay in 96-well flat-bottom microplates (EuroClone Primo multiwell plate, flat bottom, Pero, Milan, Italy), following the protocol described by Bellio et al. [38]. Tested compounds included PMB (Sigma-Aldrich), which disrupts Gram-negative outer membranes; CCCP (Sigma-Aldrich), a protonophore that collapses the proton motive force across bacterial membranes; and PaβN (VWR Avantor, Milan, Italy), an efflux-pump inhibitor that enhances intracellular antibiotic accumulation, mainly in Gram-negative bacteria. Briefly, two-fold serial dilutions of AF-Cl were performed in MHB along the abscissa, while the second drug was two-fold diluted in MHB along the ordinate (final volume/well 100 µL). The resulting checkerboard contained a total of 77 combinations of the two antibacterials. Bacterial cultures were prepared from freshly isolated colonies and suspended in sterile saline to achieve a turbidity equivalent to 0.5 McFarland (≈1.5 × 10^8^ CFU/mL). The suspension was subsequently diluted 1:150 in MHB, and 100 µL of this suspension was added to each well of the checkerboard plate yielding a final inoculum of approximately 5 × 10^5^ CFU/well (final volume/well 200 µL). Each plate included MHB alone (negative control) and MHB with bacteria in the presence of solvent (positive control). Plates were incubated at 37 ± 1 °C for 18 ± 2 h, and MICs were determined as the lowest concentrations of the compounds (alone or in combination) that completely inhibited bacterial growth.

### 4.5. Synergy Assessment Using SynergyFinder

Drug combination effects were analyzed using the SynergyFinder web application (https://synergyfinder.fimm.fi accessed on 22 April 2026), a validated platform for interactive evaluation and visualization of multidrug response data [14,15]. Dose–response matrices were obtained from the microdilution assay data, and synergy scores were calculated and interpreted according to the user guide available on the website (https://synergyfinder.aittokallio.group/synfin_docs/ accessed on 22 April 2026). When synergy scores are less than −10, the interaction between two drugs is likely to be antagonistic; from −10 to 10, the interaction between two drugs is likely to be additive; and larger than 10, the interaction between two drugs is likely to be synergistic. Similar thresholds were applied to the most synergistic area score, which represents the most synergistic 3-by-3 dose-window in a dose–response matrix.

### 4.6. Synergy Assessment Using the Fractional Inhibitory Concentration Index (FICi)

The FICi of two-drug combinations (A and B) was calculated as follows: (MIC of A in combination/MIC of A alone) + (MIC of B in combination/MIC of B alone). Interpretation thresholds were as follows: FICi ≤ 0.5, synergy; 0.5 < FICi < 4, indifference; and FICi > 4, antagonism. To investigate the directionality and nature of the interaction between the two compounds, the individual FICis were considered as separate metrics as FICA→B (MIC of B in combination/MIC of B alone) and FICB→A (MIC of A in combination/MIC of A alone) and interpreted according to [16]. Specifically, FICA→B ≤ 0.25 was interpreted as compound A promoting the activity of compound B; 0.25 < FICA→B < 2 indicated an additive/independent effect; and FICA→B = 0.5 represented the threshold between additivity and independence; conversely, FICA→B ≥ 2 was considered indicative of inhibition of compound B activity by compound A. The same interpretative framework was applied to FICB→A to evaluate reciprocal effects.

### 4.7. Synergy Evaluation by Colony-Forming Unit (CFU) Enumeration

Bacterial viability of the synergistic combinations identified in the checkerboard assay was confirmed by the CFU count. After 24 h of incubation, 100 µL of each culture—both the selected drug combinations and the corresponding monotherapy controls—were collected and serially 10-fold diluted in MHB. A total of 200 µL of the appropriate dilutions was plated in duplicate on tryptic soy agar (TSA, Liofilchem S.r.l., Roseto degli Abruzzi, Teramo, Italy) and plates were incubated at 37 °C for 18–24 h. Colonies were counted manually, and CFU/mL was calculated by considering the dilution factor. Synergy was defined as a ≥ 2 log_10_ reduction in CFU/mL at 24 h for the combination in comparison to the single most active drug, according to NCCLS guidelines (document M26-A) [39].

### 4.8. Evaluation of the Effects of GSH Depletors on AF-Cl Antibacterial Activity

The impact of BSO, DEM (VWR Avantor), and their combination on the antibacterial activity of AF-Cl was evaluated against *P. aeruginosa* ATCC 15442. BSO and DEM were employed at fixed concentrations of 20 mM and 2.5 mM, respectively—values established in preliminary assays—as the highest concentrations that exhibited no or minimal effects on *P. aeruginosa* growth when applied individually or in combination. Growth-inhibition assays were performed in 96-well microtiter plates by combining AF-Cl (1–16 µg/mL) with each GSH-depleting agent, individually or in combination. Cultures were incubated at 37 °C for 20–24 h. After incubation, OD_620_ values were measured, and data for each AF-Cl concentration were normalized by calculating the ratio between the OD_620_ of treated samples and that of the corresponding control (bacteria grown in medium supplemented with equivalent concentrations of DMSO).

In a separate set of experiments, the effects of BSO, DEM, and their combination on the antibacterial activity of AF-Cl (tested at 2 µg/mL) were assessed by quantifying viable bacteria. At the end of the incubation period, cultures were serially diluted and plated to determine CFU counts.

### 4.9. Statistical Analysis

Each experiment was performed in at least three independent biological replicates, each measured in duplicate or triplicate unless otherwise specified. GraphPad In Stat version 9.5.1 (GraphPad Software Inc., La Jolla, CA, USA) was used to assess the statistical significance of the data. One-way ANOVA followed by Tukey–Kramer’s multiple comparisons test was applied when assessing differences among three or more groups of data. A *p* value < 0.05 was considered statistically significant.

## 5. Conclusions

In conclusion, this study shows that the gold-derivative AF-Cl exhibits superior antibacterial activity compared to its parent compound AF, particularly when used in combination with membrane disruptors, efflux-pump inhibitors, or glutathione-depleting agents. These findings indicate that limited outer-membrane permeability, active efflux, and GSH-mediated detoxification collectively contribute to the intrinsic tolerance of Gram-negative bacteria toward gold-based conjugates such as AF-Cl.

Although still far from clinical translation, metalloantibiotics represent a promising and largely underexplored class of antimicrobial agents with the potential to expand the current antibiotic arsenal. Combination therapy, employing both antibiotic–antibiotic and antibiotic–non-antibiotic pairings, is considered an effective strategy against MDR pathogens [40,41]. In this context, combining AF-Cl—or next-generation derivatives optimized for improved uptake and reduced detoxification—with agents capable of overcoming the identified tolerance mechanisms may pave the way for new therapeutic options against drug-resistant *P. aeruginosa*.

## Figures and Tables

**Table 1:** MIC values of AF and its analogues against Gram-negative bacterial species.

Strain	AF		AF-Cl		AF-I		TPP-AuCl	
	µg/mL	µM *	µg/mL	µM	µg/mL	µM	µg/mL	µM
*E. coli* ATCC 25922	32	47.2	4	11.4	32–64	72.4–144.8	8	16.2
*E. coli* CI1	32	47.2	8	22.8	16	36.2	>64	>129.4
*E. coli* CV130121	>64	>94.4	16	45.7	16	36.2	>64	>129.4
*K. pneumoniae* ATCC 700603	>64	>94.4	16	45.7	32	72.4	>64	>129.4
*K. pneumoniae* UR	64	94.4	8	22.8	16	36.2	>64	>129.4
*K. pneumoniae* SV130121	64	94.4	16	45.7	16	36.2	>64	>129.4
*P. aeruginosa* ATCC 15442	64	94.4	32–64	45.7–91.4	32	72.4	>64	>129.4
*P. aeruginosa* W6	64	94.4	32	91.4	32	72.4	>64	>129.4
*P. aeruginosa* PORT19	>64	>94.4	64	182.8	64	144.8	>64	>129.4

* molecular weights applied for the µg/mL to µM calculations were as follows: AF: 678 gr/mol; AF-Cl: 350 gr/mol; AF-I: 442.03 gr/mol; and TPP-AuCl: 494.71 gr/mol.

**Table 2:** Synergistic combinations and corresponding FICi for AF-Cl and PMB.

Combinations							
*P. aeruginosa* Strains	MIC AF-Cl	MIC PMB	AF-Cl	PMB	FICi	FIC_PMB→AF-Cl_	FIC_AF-CL→PBM_
ATCC 15442	64 *	0.78	8	0.195	0.37	0.125	0.25
PORT19	64	0.195	16	0.048	0.49	0.25	0.246
W6	64	0.78	2	0.0975	0.15	0.031	0.125
			8	0.048	0.18	0.125	0.061

* numbers indicate µg/mL.

**Table 3:** Synergistic combinations and corresponding FICi for AF-Cl and CCCP.

Combinations							
*P. aeruginosa* Strains	MIC AF-Cl	MIC CCCP	AF-Cl	CCCP	FICi	FIC_CCCP→AF-Cl_	FIC_AF-CL→CCCP_
ATCC 15442	32 *	200	2	25	0.18	0.0265	0.125
			0.0625	50	0.2519	0.0019	0.25
PORT19	64	800	8	100	0.25	0.125	0.125
W6	64	800	16	200	0.5	0.25	0.25

* numbers indicate µg/mL.

**Table 4:** Synergistic combinations and corresponding FICi for AF-Cl and PaβN.

Combinations							
*P. aeruginosa* Strains	MIC AF-Cl	MIC PAβN	AF-Cl	PAβN	FICi	FIC_PAβN→AF-Cl_	FIC_AF-CL→PAβN_
ATCC 15442	32 *	100	2	6.25	0.125	0.0625	0.0625
			0.5	12.5	0.14	0.015	0.125
PORT19	64	100	4	12.5	0.18	0.0625	0.125
			1	25	0.26	0.0156	0.25
W6	64	100	4	12.5	0.18	0.0625	0.125
			2	25	0.28	0.0312	0.25

* numbers indicate µg/mL.

**Table 5:** Strains used in the study and their resistance profile.

Strain	Isolation Site	Resistance Profile ^a^
*E. coli* ATCC 25922	Reference strain	Susceptible
*E. coli* CI1	Urine	CTA, CRO, LEV, FOS, SXT
*E. coli* CV130121	Wound	AMP, AMC, PIP, TGC
*K. pneumoniae* ATCC 700603	Reference strain	AMC, CAZ, CRO, ERT, GEN, SXT
*K. pneumoniae* UR	Urine	AMP, PIP
*K. pneumoniae* SV130121	Wound	AMC, FEP, CAZ, CZA, CTB, CTA, CRO, CIP, ERT, MEM, TZP, SXT
*P. aeruginosa* ATCC 15442	Reference strain	Susceptible
*P. aeruginosa* W6	Wound	AMP, ATM, AMC, CAZ, CIP, CRO, CXM, ERT, FEP, FOS, FOX, TGC, TZP
*P. aeruginosa* PORT19	Catheter	CIP, LVX

^a^ AMC: amoxicillin/clavulanate; AMP: ampicillin; ATM: aztreonam; CAZ: ceftazidime; CIP: ciprofloxacin; CRO: ceftriaxone; CTA: ceftolozane/tazobactam; CTB: ceftibuten; CXM: cefuroxime; CZA: ceftazidime/avibactam; ERT: ertapenem; FEP: cefepime; FOS: fosfomycin; FOX: cefoxitin; GEN: gentamicin; LEV: levofloxacin; MEM: meropenem; PIP: piperacillin; SXT: trimethoprim/sulfamethoxazole; TGC: tigecycline; TZP: piperacillin/tazobactam.

## Data Availability

The raw data supporting the conclusions of this article will be made available by the authors on request.
